# Treatment of ursodeoxycholic acid with glucocorticoids and immunosuppressants may improve the long-term survival rate in primary biliary cholangitis patients

**DOI:** 10.1097/MD.0000000000031395

**Published:** 2022-11-18

**Authors:** Zi-Long Wang, Rui Jin, Mei Hao, Yan-Di Xie, Zhi-Cheng Liu, Xiao-Xiao Wang, Bo Feng

**Affiliations:** a Beijing Key Laboratory of Hepatitis C and Immunotherapy for Liver Diseases, Peking University Hepatology Institute, Peking University People’s Hospital, Beijing, China; b Medical Information Center, Peking University People’s Hospital, Beijing, China.

**Keywords:** glucocorticoid, immunosuppressant, primary biliary cholangitis, prognostic factors, ursodeoxycholic acid

## Abstract

Primary biliary cholangitis (PBC) is an autoimmune cholestatic liver disease. The clinical effectiveness of ursodeoxycholic acid (UDCA) plus glucocorticoids and/or immunosuppressants remains controversial in PBC patients. The study aimed to compare the efficacy of monotherapy and combination therapy in patients with PBC and to assess the factors affecting the efficacy. In this retrospective study, 266 patients diagnosed with PBC were divided into monotherapy group (UDCA), double therapy group (UDCA plus glucocorticoids or immunosuppressants), and triple therapy group (UDCA plus glucocorticoids and immunosuppressants) according to different treatments. Demographic characteristics, immune parameters, biochemistry profiles, and other indicators were evaluated at baseline, 6 months, and 1 year following treatment. The prognosis was evaluated using the Paris II standard. The liver transplant-free survival at 3, 5, 10, and 15 years was predicted by GLOBE score. All statistical analyses were conducted using SPSS (version 24) software (SPSS Inc, Chicago, IL). The long-term survival rate of the triple therapy group was significantly improved compared with the monotherapy group (*P* = .005). In addition, multivariate analysis showed that abnormal platelet count, alkaline phosphatase, and albumin levels were risk factors for poor response. When IgG levels were elevated but below twice the upper limit of normal, the clinical benefit was not significant compared with monotherapy (*P* > .05). Compared with monotherapy and double therapy, triple therapy may improve the long-term survival rate of PBC patients. Abnormal platelet count, alkaline phosphatase, and albumin levels were associated with a poor prognosis.

## 1. Introduction

Primary biliary cholangitis (PBC) is an autoimmune cholestatic liver disease that is related to the loss of immune tolerance of mitochondrial antigens. Subsequent humoral and cellular immunity and environmental and genetic factors jointly promote the occurrence of PBC.^[1]^ It is characterized by the non-suppurative destruction of small bile ducts,^[2]^ which in turn may develop into hepatic fibrosis or even cirrhosis. In the past, PBC was considered a disease with low prevalence. However, with the increased understanding of the disease and the improvement of detection technology, the prevalence of PBC has been increasing recently, ranging from 21.7 to 39.2 per 100,000 people from 2004 to 2014.^[3]^

Ursodeoxycholic acid (UDCA) is a first-line drug approved for the treatment of PBC, and the therapy derives benefit by accelerating bile acid enterohepatic circulation, stabilizing the biliary HCO_3_^-^ umbrella, anti-apoptosis, and anti-inflammatory.^[4]^ In an international multicenter cohort study, the 10-year cumulative liver-free survival of patients treated with UDCA was significantly higher than that of untreated patients, and the benefits were significant regardless of sex, age, or disease stage.^[5]^ Unfortunately, 40% of patients showed a suboptimal response to UDCA, and the liver transplant-free survival rate of these patients is significantly lower than in patients who respond well to UDCA.^[6]^

Obeticholic acid and fibrates are usually proposed as second-line drugs.^[7–9]^ However, for patients with poor UDCA response, the combined treatment can significantly improve biochemical and itching symptoms, but large-scale prospective studies of long-term efficacy are still lacking.

Many studies showed some contradictory results for the efficacy of glucocorticoids and immunosuppressants to PBC. For PBC patients, glucocorticoids and immunosuppressants are not recommended, but for PBC-AIH overlap syndrome (OS) patients, combining immunosuppressive therapy with UDCA is recommended. Many patients have certain characteristics of AIH but do not meet the diagnostic criteria of OS. To find people who are sensitive to combination therapy, we compared the therapeutic effects of UDCA as monotherapy, double therapy (UDCA combined with glucocorticoids or immunosuppressants), and triple therapy (UDCA combined with glucocorticoids and immunosuppressants) on PBC patients. We subsequently analyzed the factors influencing the efficacy of the treatment response and evaluated the characteristics of patients with poor responses to UDCA as a basis for further work on PBC therapy.

## 2. Materials and methods

### 2.1. Patient population

Between January 2013 and December 2018, 797 patients from the Peking University People’s Hospital Clinical Data Repository.

#### 2.1.1. Inclusion criteria.

The diagnosis of PBC was based on the AASLD 2018 Practice Guidance on PBC^[10]^; UDCA and immunosuppressive drugs were initially treated in newly diagnosed PBC patients in the hospital.

#### 2.1.2. Exclusion criteria.

PBC-AIH (OS), deficiency of complete clinical data, UDCA combined with other drugs such as fibrates or less than 6 months.

The study was carried out according to the guidelines of the Helsinki Declaration and was approved by the ethics committee of Peking University People’s Hospital. All clinical data were collected following Peking University People’s Hospital Ethics Review Committee approval (2019PHB279-01). No informed consent was required because the data are anonymized.

### 2.2. Data collection

The following data at baseline, 6 months, and 1 year after treatment were collected from the Clinical Data Repository: demographic characteristics: gender and age; immune parameters: immunoglobulin A (IgA), IgG, IgM, AMA-M2; biochemistry profiles: alanine aminotransferase (ALT), aspartate aminotransferase (AST), gamma-glutamyl transferase (GGT), alkaline phosphatase (ALP), albumin (ALB), total bilirubin (TBIL), and plateles (PLT); the type and dosage of medicine taken by the patient. In addition, the proportion of patients with cirrhosis at baseline was recorded according to a liver biopsy and radiological evidence. The comorbidities were classified according to the International Disease Code (ICD-10).

### 2.3. Prognosis judgment

The Paris II criteria and GLOBE score were used to evaluate the 1-year efficacy and liver transplant-free survival rate. The data from each patient were obtained 1 year after the beginning of therapy.

The Paris II criteria consisted of ALP and AST ≤ 1.5 upper limits of normal, with a normal bilirubin level.^[11]^ The GLOBE score of each patient was calculated based on their age, levels of TBIL, ALP, ALB, and PLT after 1 year of UDCA monotherapy. The GLOBE score^[12]^ is a predictor of liver transplant-free survival at 3, 5, 10, and 15 years. The score and the liver transplant-free survival of each patient was also calculated using age and other biochemistry profiles after the 1 year of combination therapy.

### 2.4. Statistical analysis

Demographics and baseline laboratory test results are reported using mean (standard deviation) and median (interquartile range) values for continuous variables and numbers and percentages for categorical variables. The Kolmogorov-Smirnov method was used to test the normality of measurement data. The *t* test or one-way ANOVA method was used to compare samples or multiple samples of normal distribution data. If the variance was uneven, the Welch variance analysis method was adopted. Two rates or two constituent ratios were compared by the chi-square test. A logistic regression model was used to analyze the risk factors and odds ratio (OR) associated with poor prognosis. All analyses were considered exploratory and were performed using SPSS 24.0 (IBM Corp., Armonk, NY) and GraphPad Prism 7 (GraphPad Software, La Jolla, CA), with *P* < .05 considered significant.

## 3. Results

### 3.1. Study population and baseline characteristics

**Figure 1.**
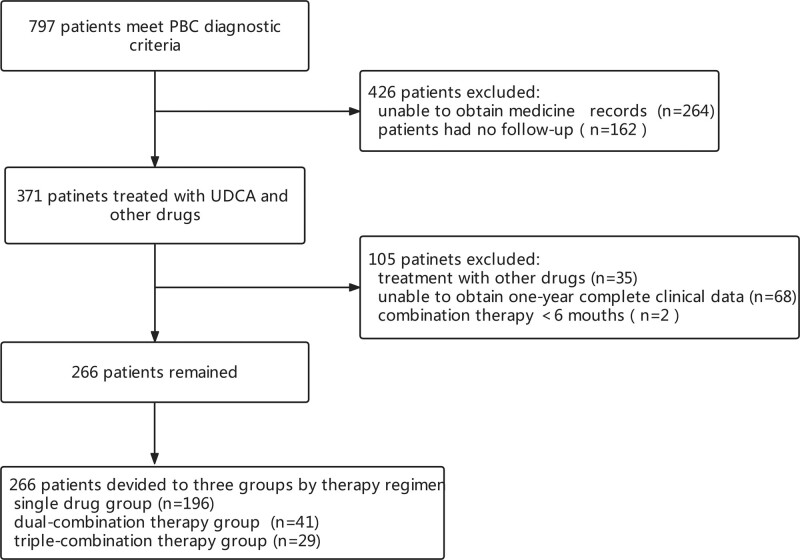
Flowchart of the patient selection process.

Among the 797 patients who met PBC diagnostic criteria, 264 patients were excluded because UDCA medication records could not be found, and 162 patients were also excluded owing to the lack of follow-up examination. Among the remaining 371 patients, 35 patients were excluded owing to treatment with other drugs, and 68 patients were excluded because complete clinical data could not be obtained for 1 year. In addition, two patients who had been treated with combination therapy for less than 6 months were excluded to reduce the possible effect of lower treatment duration. Finally, 266 patients were included in the study: 196 patients in the single-drug group (UDCA monotherapy), 41 patients in the dual-combination group (UDCA combined with glucocorticoids or immunosuppressants), and 29 patients in triple-combination therapy (UDCA combined with glucocorticoids and immunosuppressants) (Fig. 1). The medications used by the combination therapy group at the time of therapy were analyzed. Prednisone and methylprednisolone were the main glucocorticoids, while cyclosporine, azathioprine, and mycophenolate mofetil dispersible tablets were the main immunosuppressants.

The baseline demographics and clinical features of the enrolled patients are shown in Table 1. Of the 266 enrolled patients who were initially diagnosed with PBC, 233 (87.6%) were female and 33 (12.4%) were male patients. These 266 patients were divided into three groups (groups A, B, and C) according to the treatment regimen. The average age of the patients was 58.35 ± 11.02 years. There were no statistically differences in demographics; immunological indexes such as IgA, IgG, IgM, and AMA-M2; biochemical indexes such as ALT, AST, GGT, ALP, ALB, TBIL, and PLT; and the proportion of baseline cirrhosis between the 3 groups (*P* > .05). The patients with cirrhosis were identified by pathological assessment or imaging techniques, such as ultrasound and MRI.

**Table 1:** Baseline characteristics of PBC patients.

	All patients (n = 266)	Group A (n = 196)	Group B (n = 41)	Group C (n = 29)	*P* value
Demographic characteristics					
Age	58.35 ± 11.02	58.95 ± 11.19	58.78 ± 10.72	53.83 + 9.46	.058
Sex					
Female	233 (87.6%)	170 (86.7%)	37 (90.2%)	26 (89.6%)	.758
Male	33 (12.4%)	26 (13.3%)	4 (9.8%)	3 (10.4%)	
Immune parameters					
IgA (g/L)	3.14 (0.35–20.30)	3.10 (0.35–20.3)	3.46 (0.82–16.70)	3.36 (1.03–9.66)	.986
IgG (g/L)	16.50 (1.87–61.10)	16.40 (1.87–61.1)	15.55 (2.12–26.10)	18.90 (2.92–46.10)	.232
IgM (g/L)	2.90 (0.456–30.30)	2.92 (0.45–30.30)	2.99 (0.83–10.50)	2.82 (0.65–6.96)	.879
AMA-M2	601.82 (34.13–1033.36)	520.32 (34.13–1033.36)	644.39 (45.61–934.07)	658.89 (514.19–803.59)	.705
Biochemistry profiles					
ALT (U/L)	38 (8–2010)	40.5 (8–2010)	45 (16–590)	28 (13–784)	.883
AST (U/L)	43 (15–1370)	43 (16–1370)	51 (19–198)	36.5 (15–544)	.691
GGT (U/L)	129 (8–1889)	130.5 (8–1889)	129 (16–1000)	106 (25–472)	.736
ALP (U/L)	142 (43–1496)	139.5 (43–1496)	185 (58–908)	147 (48–615)	.216
ALB (g/L)	42.30 (24.90–53.30)	42.55 (24.90–53.30)	41.90 (25.60–48.50)	42.20 (30.70–50.40)	.731
TBIL (μmol/L)	15.00 (4.70–150.10)	14.90 (6.00–138.70)	14.80 (7.90–150.10)	18.00 (4.70–49.80)	.080
PLT (10^9^/L)	183 (6–502)	183 (14–502)	181 (11–327)	183 (6–370)	.646
Liver cirrhosis	35 (13.16%)	23 (11.73%)	4 (9.76%)	8 (27.59%)	.140

*P*-value represents a comparison between three groups.

Age is expressed as mean ± SD. Other characteristics are shown as the median (minimum and maximum). Statistical analysis was performed using one-way ANOVA.

ALB = albumin, ALP = alkaline phosphatase, ALT = alanine amino-transferase, AMA-M2 = anti-mitochondrial antibody-M2, AST = aspartate aminotransferase, GGT = γ-glutamyl transpeptidase, Group A = UDCA monotherapy group, Group B = dual-combination therapy group, Group C = triple-combination therapy group, IgA = immunoglobulin A, IgG = immunoglobulin G, IgM = immunoglobulin M, PBC = primary biliary cholangitis, PLT = platelets, TBIL = total bilirubin.

### 3.2. Effect of combination therapy on the GLOBE score

**Figure 2.**
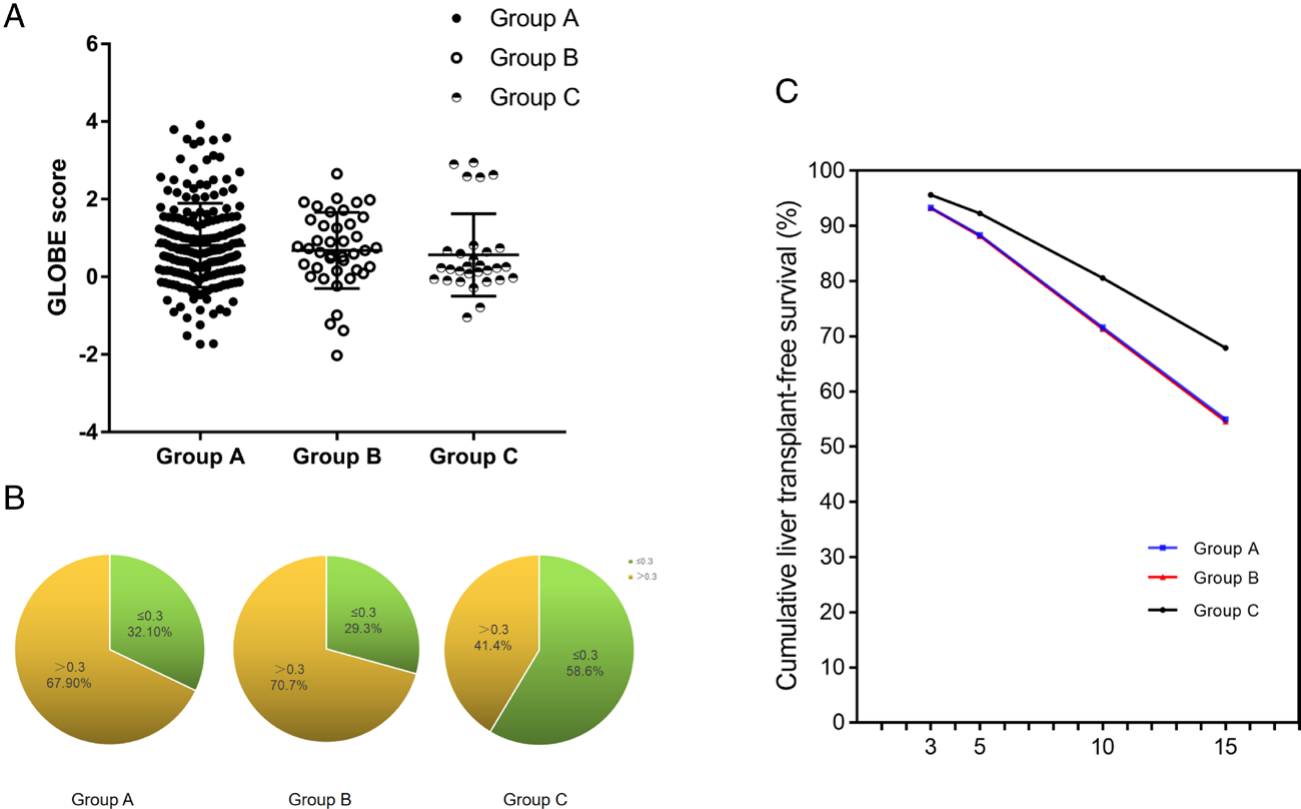
Survival analysis of the different groups. (A) The GLOBE score of 3 groups, data are expressed as the mean ± SD. (B) Group with GLOBE score >0.3, the proportion of 3 groups. (C) Transplant-free survival curves of different groups. Data are expressed as the IQR. The blue line shows group A, the red line show group B, and the black line show group C. Group A: UDCA monotherapy group; Group B: dual-combination therapy group; Group C: triple-combination therapy group. IQR = interquartile range, SD = standard deviation, UDCA = ursodeoxycholic acid.

The effect of combination therapy on the estimated survival rates calculated using the GLOBE score is shown in Figure 2A. The mean GLOBE score from groups A, B, and C were 0.82 ± 1.08, 0.68 ± 0.98, and 0.57 ± 1.06. A downward trend can be observed, but the difference was not statistically significant (*P* > .05). A GLOBE score greater than 0.3 indicates a significant reduction in long-term survival compared with that of a matched general population. The three groups were divided into two parts according to a GLOBE score greater than 0.3. Notably, the proportion of people with a GLOBE score greater than 0.3 in the triple-combination therapy group was significantly reduced (Fig. 2B). Transplant-free survival curves of different groups were calculated by the GLOBE score. The survival S(t) for any given patients was then calculated by S(t) = S0(t)exp(GLOBE score) (Fig. 2C). The results showed that there were significant statistical differences between the triple-combination therapy group and single-drug group (*P* < .01), while there were no statistical differences between the dual-combination therapy group and the single drug group (*P* = .72) (Table 2). This observation suggests that PBC patients treated with triple therapy may have a higher long-term survival rate than UDCA monotherapy.

**Table 2:** Comparison of long-term survival rate of different treatment groups.

	GLOBE score ≤ 0.3	GLOBE score > 0.3	*P* value
Group A (n = 196)	63	133	
Group B (n = 41)	12	29	.719^a^
Group C (n = 29)	17	12	.005^b^

According to the GLOBE score greater than 0.3, the number of patients in group C was significantly reduced. (a) means compared with group A; (b) means compared with group B. Statistical significance was performed by chi-square test.

Group A = ursodeoxycholic acid monotherapy group, Group B = dual-combination therapy group, Group C = triple-combination therapy group.

### 3.3. Response of PBC patients with high igg to combination therapy

Among 79 patients with higher IgG levels than normal, 74 patients had more than 1 upper limit of normal (ULN) and 5 had more than 2 ULN. According to the Paris II standard, the 1-year response rate of PBC patients with high IgG was estimated. Compared with group A, the clinical benefit of UDCA combined with glucocorticoids and/or immunosuppressants was not obvious compared with that of UDCA monotherapy (*P* > .05) (Table 3). Assessment using Welch analysis of variance showed no significant difference in GLOBE score among patients with higher IgG levels from the three groups.

**Table 3:** Response ratios of patients with high IgG in different treatment groups.

High levels of IgG	Response well	Response poor	*P* value
Group A (n = 55)	29	26	
Group B (n = 12)	8	4	.379
Group C (n = 12)	7	5	.724

Compared with group A, group B and group C did not show significant differences for the response rate in the patients with high IgG level.

Group A = ursodeoxycholic acid monotherapy group, Group B = dual-combination therapy group, Group C = triple-combination therapy group, IgG = immunoglobulin G.

### 3.4. Univariate and multivariate analysis of patient response to treatment

Among 266 PBC patients, there were 186 (69.9%) good responders and 80 (30.1%) poor responders. Univariate analysis showed that compared with patients with a positive response, the patients with poor response consist of a higher proportion of males, have higher levels of IgG, GGT, ALP, and TBIL (*P* < .05), and have lower levels of platelet count and ALB (*P* < .05). Based on multivariable logistic regression analysis, the statistically significant indicators and therapy methods were included. The results showed that higher ALP (OR = 1.01; *P* < .01; 95% CI: 1.01–1.02), lower PLT (OR = 0.99; *P* < .01; 95% CI: 0.99–1.00) and ALB levels (OR = 0.85; *P* < .01; 95% CI: 0.77–0.92) were associated with poor response (Table 4).

**Table 4:** Univariate and multivariate analysis of treatment response in all patients.

	Univariable OR		Multivariable OR	
	OR (95% CI)	*P* value	OR (95% CI)	*P* value
Group		*P* = .123		
Demographic characteristics				
Age		*P* = .851		
Sex				
Female	0.005 (0.165–0.731)	*P* = .005		*P* = .099
Male				
PLT (10^9^/L)	0.993 (0.989–0.997)	*P* < .001	0.992 (0.987–0.998)	*P* = .009
Immunological indicators				
IgA (g/L)		*P* = .194		
IgG (g/L)	1.055 (1.012–1.100)	*P* = .013		*P* = .387
IgM (g/L)		*P* = .987		
IgG:IgM		*P* = .978		
Biochemical indicators				
ALT (U/L)		*P* = .757		
AST (U/L)		*P* = .399		
GGT (U/L)	1.003 (1.002–1.006)	*P* < .001		*P* = .349
ALP (U/L)	1.010 (1.007–1.013)	*P* < .001	1.010 (1.005–1.015)	*P* < .001
ALB (g/L)	0.841 (0.797–0.887)	*P* < .001	0.846 (0.774–0.924)	*P* < .001
TBIL (μmol/L)	1.039 (1.021–1.057)	*P* < .001		*P* = .573

The multivariate model was adjusted for sex, PLT, IgG, GGT, ALP, ALB, and TBIL.

ALB = albumin, ALP = alkaline phosphatase, ALT = alanine amino-transferase, AST = aspartate aminotransferase, CI = confidence interval, GGT = γ-glutamyl transpeptidase, IgA = immunoglobulin A, IgG = immunoglobulin G, IgM = immunoglobulin M, PLT = plateles, OR = odds ratio, TBIL = total bilirubin.

## 4. Discussion

Prior studies have indicated that UDCA combined with glucocorticoid or immunosuppressant have more effective treatment outcomes on PBC patients,^[13,14]^ while others took an opposing view. The cause of diverse results may be the small number of patients, the different observation times, and races. Recently, a study showed that UDCA combined with budesonide has little benefit for patients with poor UDCA response,^[15]^ these inconsistent conclusions pose a difficulty for clinicians. Therefore, we conducted this retrospective study in PBC patients receiving UDCA monotherapy and combination therapy.

To perform this study, it was necessary to estimate the outcome of treated patients. We used different models to predict the prognosis as accurately as possible. According to the Paris II standard, patients undergoing UDCA treatment for 1 year were evaluated for ALP, AST, and bilirubin levels, which were divided into two groups: the positive response group and the poor response group. However, the prognosis of each patient could not be predicted.^[11]^ Therefore, we used the GLOBE score system following UDCA treatment for 1 year to estimate the survival time without liver transplantation.^[12]^ The clinical value of this score in Chinese patients has also been verified.^[16]^

A downward trend in some biochemical indicators such as ALT, AST, and GGT was observed for the combination therapy group, although 1-year response rate between the three groups based on the Paris II was not statistically significant. The long-term survival rate of patients was evaluated depending on whether the GLOBE score was greater than 0.3, where a GLOBE score >0.3 means that the long-term survival rate is lower than the matched general population.^[12]^ The results show that triple therapy may improve the long-term survival rate of patients than the other two groups, although it has few benefits in efficacy for a year. The inconsistent results may be due to the following reasons.

On the one hand, triple therapy using drugs with different pharmaceutical mechanisms leads to being more effective. Glucocorticoids are widely used in inflammatory diseases, and their biological effects are mediated by glucocorticoid receptors antagonizing proinflammatory transcription factors.^[17]^ In recent years, it has also been found that glucocorticoids were effective in the treatment of cholestatic liver diseases, which may be related to the inhibition of bile acid synthesis through inhibiting the expression of CYP7A1, the rate-limiting enzyme of bile acid synthesis.^[18]^ Besides, glucocorticoids can also increase ileal bile acid sodium transporter expression to stimulate the absorption of bile acid in liver cells and basolateral bile transporter expression to increase liver bile acid intake.^[18,19]^ Immunosuppressants are commonly used in the treatment of autoimmune diseases via reducing the number of lymphocytes and regulating the function of lymphocytes.

On the other hand, PBC has unique characteristics. Although some scholars believe that PBC is caused by a secretory defect of bicarbonate produced by the biliary system,^[20]^ it seems to display more autoimmune features. Floreani reported statistics on 361 cases of PBC patients during 1975 to 2012 and showed that 221 patients (61.2%) had at least one type of extrahepatic autoimmune disease.^[21]^ A study from China also showed that about half of the AMA-positive population had autoimmune diseases.^[22]^

Thus, treating comorbid diseases in PBC patients may improve the long-term survival rate. However, this hypothesis still needs further verification.

Based on our results, we conclude that triple therapy may improve the long-term survival rate of PBC patients. Advanced fibrosis and compensated cirrhosis may be an important reason for the effect of the therapy. The prognosis of compensated cirrhotic patients at different group responded similarly compared with non-cirrhotic patients. Therefore, the influence of cirrhosis could be excluded. One could argue that the patients with combination therapy do not have PBC, but PBC-AIH OS or variant syndrome. However, only four patients with an IgG level of more than 2 ULN and ALT level of more than 5 ULN satisfied OS criteria according to Paris Standard.^[23]^ The International Autoimmune Hepatitis Group recommends the use of variant syndrome to describe patients with two disease characteristics.^[24]^ If PBC patients have abnormal examination results, such as elevated IgG and transaminases, variant syndrome should be suspected.^[25]^ Compared with the increase in transaminases, the increase in IgG level may be more specific, so the IgG index was selected for analysis.

According to the Paris standard, IgG higher than 2 ULN is a diagnostic indicator for variant syndrome.^[23]^ There were 74 patients with IgG levels higher than 1 ULN and 5 patients with more than 2 ULN. Compared with the UDCA monotherapy group, there was no significant difference in 1-year efficacy with combination therapy for PBC patients with high IgG, suggesting no clinical benefit of combined therapy compared with UDCA monotherapy treatment. Although the variant syndrome population may be more sensitive to immunosuppressive therapy, close attention should be paid to the IgG level of patients to prevent the over-diagnosis of variant syndrome and avoid unnecessary immunosuppressive treatment. Since the number of cases greater than 2 ULN is too small, no statistical analysis was conducted.

Early detection of patients with poor response to UDCA is important. Therefore, the factors that affect the efficacy of PBC patients require in-depth research. Previous studies have shown that age, sex,^[26]^ and biochemical indicators^[27]^ are the influencing factors of UDCA treatment response. According to the Paris II standard, patients were divided into a positive response group and a poor response group. The baseline clinical data from two groups were compared, the poor response group had a higher percentage of male patients; the levels of IgG, GGT, ALP, and TBIL were higher; and the levels of ALB and PLT were lower. Cholestasis is the key link in the pathogenesis of PBC. GGT and ALP are the enzyme markers of bile duct injury and TBIL reflects the degree of cholestasis. An increase in the above indicators suggests that the degree of cholestasis is more serious, while a decrease in platelet and ALB levels can partly reflect the decline in liver function, which may explain the poor response.

The above-mentioned risk factors and different treatment regimens were included in the multivariate logistic regression analysis. We found that high ALP and low ALB and PLT were the risk factors affecting the response to treatment. Our patients have no statistical differences in age as opposed to a previous study, which may be because disease symptoms are neglected in young patients, leading to the bias of the included population and the small number of patients. Although the elevated level of IgG has not met the diagnostic criteria of variant syndrome, this study suggests that the elevated IgG level is related to poor response and may be related to the characteristics of AIH, which needs further verification.

Our study has three main limitations. First, the number of patients treated with combination therapy was small. Only when patients visit other departments or have comorbid diseases can they use glucocorticoids or immunosuppressants. Second, we used the Paris II standard and GLOBE score to evaluate the prognosis of patients receiving combined treatment, which was only verified in PBC patients who received UDCA treatment for 1 year. However, the researchers found that the actual survival rate of UDCA combined with bezafibrate was higher than that estimated by the GLOBE score.^[7]^ Whether UDCA combined with glucocorticoid or immunosuppressive therapy also underestimated the survival rate needs further study.

Finally, the analysis was a retrospective study, making it difficult to obtain the data on adverse effects and liver pathology, which is very important for the accurate and comprehensive evaluation of the efficacy of the combination therapy. As a result, the level of evidence is relatively low.

## 5. Conclusions

Compared with UDCA monotherapy, UDCA combined with glucocorticoids and immunosuppressants may improve the long-term survival rate of patients. UDCA combined with glucocorticoid or immunosuppressive agents cannot improve the response rate of patients with high IgG below twice the upper limit of normal value. Abnormal platelet count, ALP, and ALB levels were associated with a poor prognosis.

## Author contributions

**Conceptualization:** Bo Feng.

**Data curation:** Rui Jin, Mei Hao, Zhi-Cheng Liu.

**Formal analysis:** Zi-Long Wang.

**Methodology:** Yan-Di Xie, Xiao-Xiao Wang.
